# Hill-Sachs Lesion: Diagnosis, Classification, and Treatment

**DOI:** 10.1055/s-0045-1809339

**Published:** 2025-07-10

**Authors:** Marcel Jun Sugawara Tamaoki, Artur Yudi Utino, Renato Aroca Zan, Fabio Teruo Matsunaga, Nicola Archetti Netto

**Affiliations:** 1 Discipline of Hand and Upper Limb Surgery, Shoulder and Elbow Group, Department of Orthopedics and Traumatology, Escola Paulista de Medicina, Universidade Federal de São Paulo, São Paulo, SP, Brazil

**Keywords:** humeral fractures, joint instability, orthopedic procedures, shoulder dislocation, fratura do úmero, instabilidade articular, luxação do ombro, procedimentos ortopédicos

## Abstract

A lesão de Hill-Sachs é uma condição frequentemente associada à instabilidade anterior do ombro, que desempenha um papel crucial nos resultados de seu tratamento The Hill-Sachs lesion is a condition frequently associated with anterior shoulder instability, which plays a crucial role in the outcomes of its treatment. It is characterized by a posterior compressive fracture in the humeral head caused by an impingement against the anterior rim of the glenoid cavity during dislocation. A thorough understanding of this lesion is essential to support clinical decisions and choose the most appropriate treatment. Advances in imaging modalities, such as magnetic resonance imaging and computed tomography, enabled lesion identification with greater precision and its classification per depth, location, and volume, resulting in a detailed assessment of its role in shoulder instability. The present article reviews the main classifications, diagnostic methods, and treatment options to provide orthopedists with a comprehensive and updated view of the strategies to promote better functional outcomes and minimize the risk of instability recurrence.

## Resumo

A lesão de Hill-Sachs é uma condição frequentemente associada à instabilidade anterior do ombro, que desempenha um papel crucial nos resultados de seu tratamento. É caracterizada por uma fratura compressiva posterior na cabeça do úmero, que resulta do impacto contra a borda anterior da cavidade glenoidal durante um episódio de luxação. A compreensão aprofundada dessa lesão é essencial para embasar decisões clínicas e para a escolha do tratamento mais adequado. Com os avanços nas modalidades de imagem, como a ressonância magnética e a tomografia computadorizada, tornou-se possível identificar a presença da lesão com maior precisão e classificá-la de acordo com sua profundidade, localização e volume, o que possibilita uma avaliação mais detalhada do seu papel na instabilidade do ombro. Este artigo revisa as principais classificações, métodos de diagnóstico e opções de tratamento, com o objetivo de fornecer ao ortopedista uma visão abrangente e atualizada das estratégias que promovem melhores desfechos funcionais e minimizam o risco de recorrência da instabilidade.

## Introduction

Although the Hill-Sachs (HS) lesion is widely known, the literature still debates its contribution to the development of shoulder instability and its implications in the surgical or conservative treatment of patients with this condition.

The first description of shoulder dislocation dates to ancient Egypt, around 3,000 BC, in the Edwin Smith papyrus. Malgaigne was the first author to mention a humeral head lesion in 1855. In 1940, 2 radiologists, Harold Arthur Hill and Maurice David Sachs, described and named the lesion. 1 2 The HS lesion is a depression of the humeral head in the posterolateral region in the presence of an anterior shoulder dislocation. This compression fracture occurs due to the impingement of the humeral head spongy bone against the anterior cortex of the glenoid cavity. 3 The HS lesion is associated with bone or labral involvement on the anterior face of the glenoid cavity and may contribute to shoulder instability. 4

Current data indicate that shoulder dislocation mainly affects young and active populations, generating concern about its socioeconomic implications. 5

The incidence of HS lesions ranges from 40% to 90% in anterior shoulder dislocations and reaches up to 100% in recurrent dislocations. 3 4 6 It is worth noting that an HS lesion rarely occurs in isolation, reinforcing the concept of bipolar injury (that is, with scapular glenoid cavity involvement), which is present in 63% of the cases. 7 8

Arm positioning at the time of dislocation is relevant, since the location and inclination of the resulting HS lesion affect shoulder stability. A dislocation occurring with the shoulder in abduction presents a greater risk of engagement (HS lesion fitting into the anterior glenoid rim). 9 More extensive HS lesions, especially in medial positions, 4 also increase the risk of instability due to reduced contact of the humeral head with the articular surface of the glenoid cavity.

## History and Clinical Picture

Patients with HS lesions typically complain of instability. They may present a history of shoulder pain, which worsens in joint abduction or hyperextension, 3 or signs such as crepitation and clicking during movement. The potential for a new dislocation increases with the number of episodes and the bone defect size. A positive apprehension test with lower degrees of abduction (mid-range) presents a higher association with glenoid cavity defects. In contrast, a positive result at the end of abduction and lateral rotation (end range) results from HS lesions. 4

To date, there is no description of a specific propaedeutic maneuver to evaluate HS lesions. A routine physical examination should assess shoulder instability, including an evaluation of generalized ligamentous laxity (Beighton criteria), and performance of the sulcus, Gagey, apprehension, surprise, and relocation tests. 10

Other tests include the hyperextension–internal rotation (HERI) test 11 and the bone apprehension test. 12 There is still controversy in the literature about the sensation of instability in lower degrees of abduction (bone apprehension test) in detecting significant bone loss in the glenoid cavity or humerus (HS). 12 13 It is crucial to confirm the finding of dislocation/instability on physical examination under anesthesia.

## Imaging Tests

**Fig. 1:**
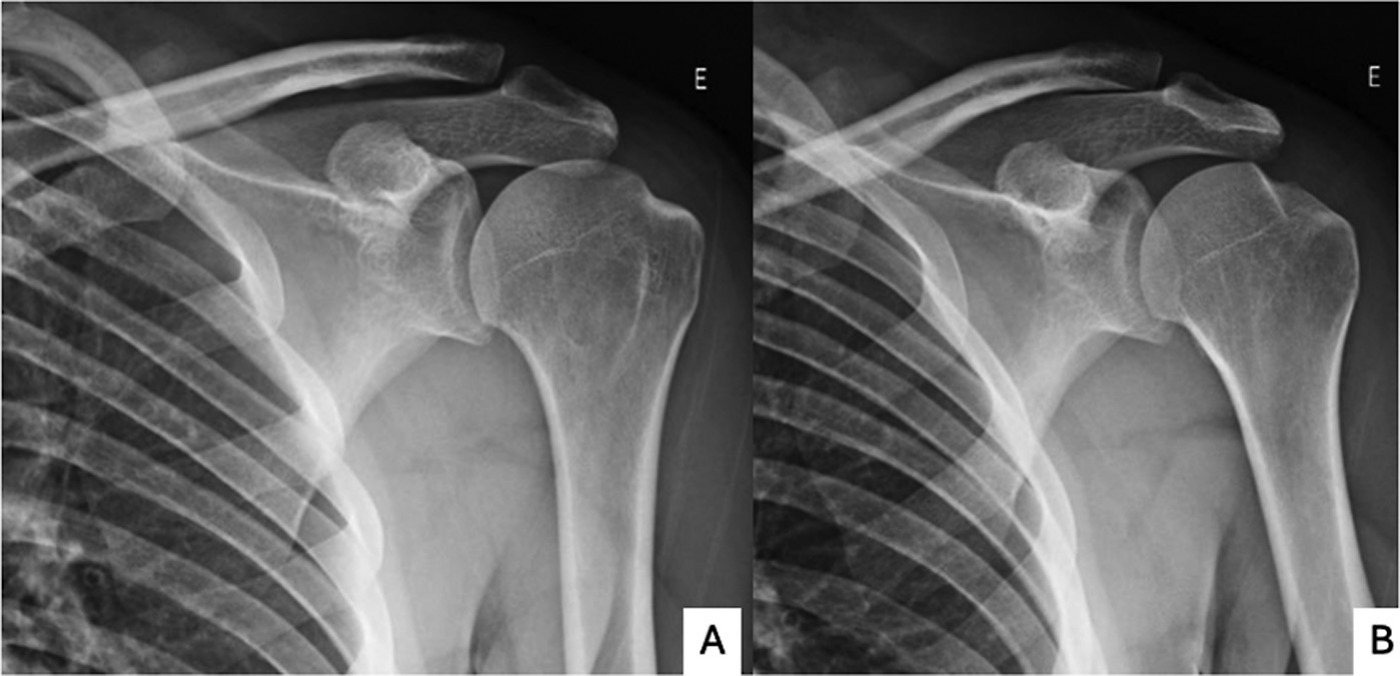
Radiographs of the shoulder with a Hill-Sachs (HS) lesion in true anteroposterior (AP), AP, axillary lateral, and scapular lateral views.

The initial investigation routinely uses shoulder radiographs in classic anteroposterior (AP), true AP, lateral, lateral scapular, and lateral axillary views ( Fig. 1 ).

The specific radiographic investigation of the humeral bone defect from an HS lesion can use the following radiographic views: 3

**Fig. 2:**
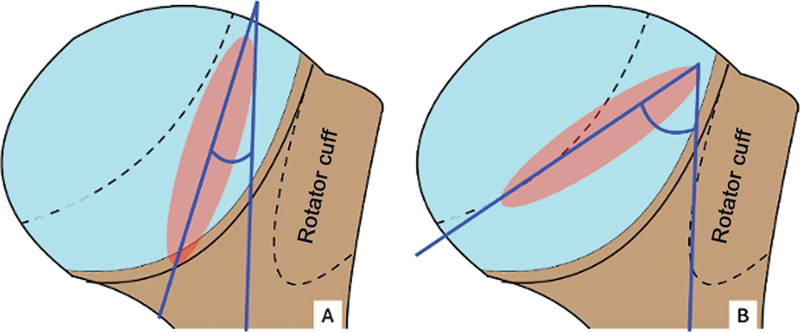
( **A** ) Anteroposterior radiograph with lateral rotation, not showing the HS lesion; ( **B** ) AP radiograph with slight medial rotation, showing the HS lesion.

- Stryker notch: medial humeral head rotation to highlight the posterolateral defect.
- Garth view: AP view of the shoulder in the scapular plane with a 45° caudal inclination of the radius.
- AP with medial rotation: this view can reveal the HS lesion size, depth, and orientation in the posterolateral region of the humeral head. 14 Often, the AP radiographic view with lateral rotation does not show the lesion, only a bone rarefaction medial to the greater tubercle 1 15 ( Fig. 2 ).
- Modified Didier: in this view, the patient is in ventral decubitus with the back of the hand resting on the posterior iliac crest, the elbow in flexion, and the radius at a 45° angle to the ground in the direction of the humeral head. 16

Radiographs have low interobserver reliability and cannot provide enough data for preoperative planning, 17 since up to 60% of bone defects may be overlooked in cases using this method alone for analysis.

**Fig. 3:**
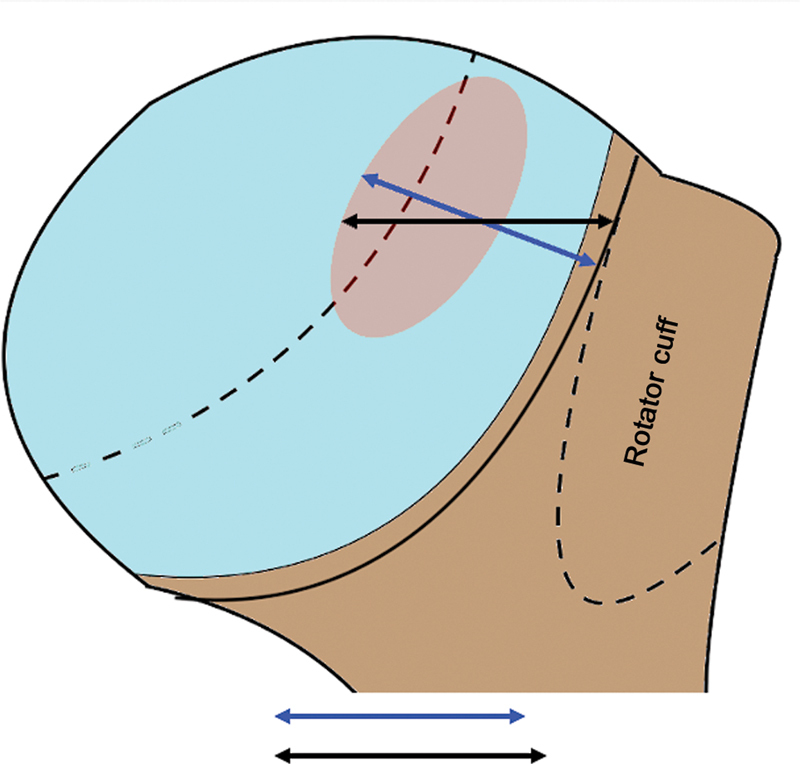
Computed tomography (CT) scans showing the HS lesion. Two-dimensional images in the axial, sagittal, and coronal sections. Three-dimensional images in the lateral and posterior views.

Computed tomography (CT) and magnetic resonance imaging (MRI) are complementary imaging methods ( Figs. 3 4 ) that are more sensitive than radiographs in the detection of HS lesions. 14

**Fig. 4:**
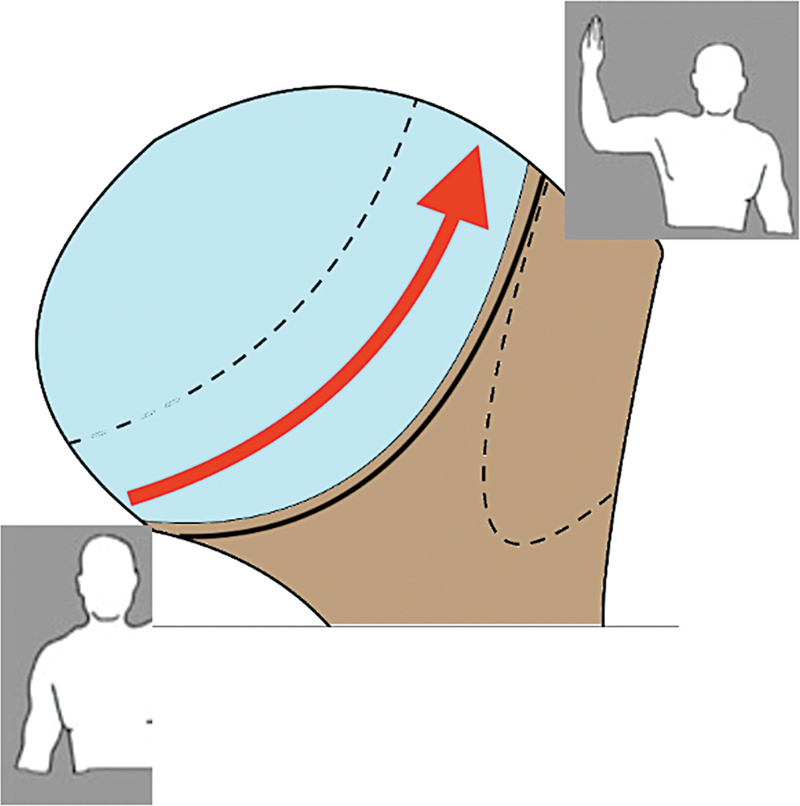
T2-weighted magnetic resonance imaging (MRI) scans showing the HS lesion the in coronal, sagittal, and axial sections.

It has been proven that there are differences in defect measurements in two- and three-dimensional (3D) images. Computed tomography with t3D reconstruction (3D-CT) is the gold standard to quantify the HS defect. 7 17 The measurements are made in slices perpendicular to the bone defect, but they can vary. 18 19

**Fig. 5:**
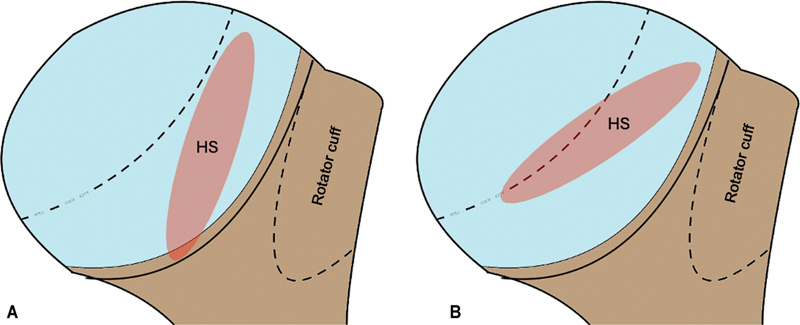
( **A** ) The smaller angle presents a lower engagement risk. ( **B** ) A larger angle presents a higher engagement risk. We considered that the HS lesion was the same size in **A** and **B** .

Three-dimensional CT defines the HS lesion angulation per the line passing deep to the injury dent and the longitudinal axis of the humeral diaphysis ( Fig. 5 ). 17 The higher the angulation, the greater the risk of engagement.

**Fig. 6:**
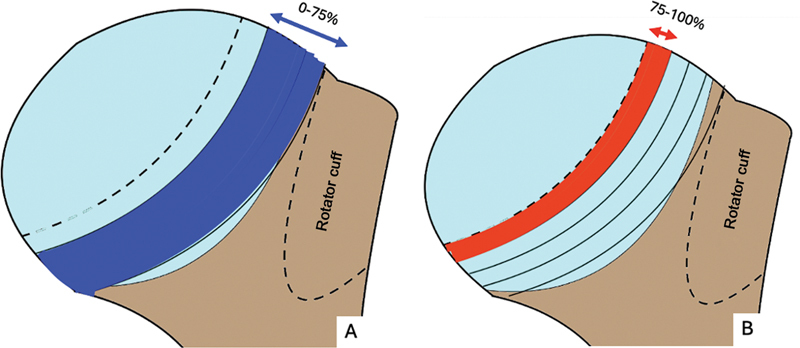
Magnetic resonance imaging scan evaluated in the axial section (black arrow); three-dimensional CT scan evaluated perpendicularly to the defect (blue arrow). Note that the black arrow is larger than the blue arrow, which may overestimate the defect and change the case management.

The use of MRI with 3D reconstruction (3D-MRI) is growing. Studies 20 21 have reported equivalent accuracy in measuring the defect of the HS lesion with 3D-CT and 3D-MRI. Three-dimensional MRI presents the advantage of not using ionizing radiation, and it has better diagnostic potential for soft tissue injuries, such as in the evaluation of rotator cuff attachment. In 3D-CT, this evaluation can result in high variability and low interobserver agreement due to the difficulty in visualizing this structure. The disadvantages of the reconstruction methods include their high costs and limited accessibility. It is worth highlighting that measuring the size of the HS lesion with two-dimensional CT or MRI increases the likelihood of obtaining an overestimated measurement (diagonally), which increases the chance of classifying the lesion as off-track and may influence the choice of the surgical technique ( Fig. 6 ).

## Differential Diagnoses

It is easier to diagnose HS lesions by associating the patient's clinical history with imaging tests. However, it is critical to remember that some conditions can cause bone erosions in the humeral head, simulating HS lesions, such as ankylosing spondylitis, rheumatoid arthritis, septic arthritis, hyperparathyroidism, hydroxyapatite storage disease, malignant tumor, or benign cysts. 14

## Classification

The classification of HS lesions can follow the arthroscopic visualization criteria described by Calandra, 7 which rely on quantifying the defect depth. A grade-1 lesion affects the articular cartilage alone; a grade-2 lesion extends to the subchondral bone; and a grade-3 lesion presents a sizable subchondral defect. 16 However, its clinical applicability is limited.

**Fig. 7:**
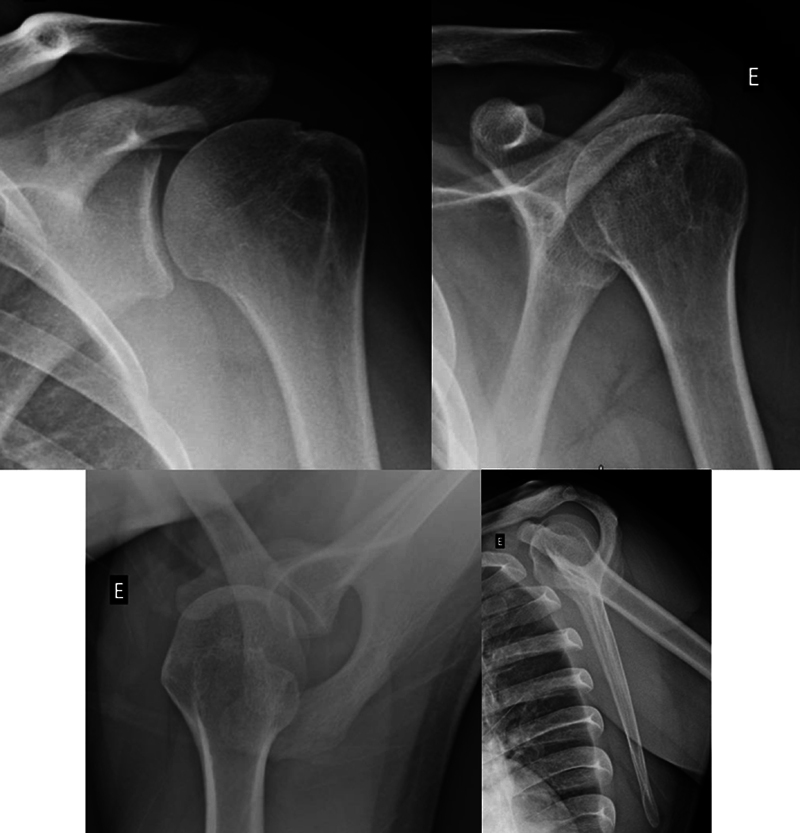
The arrow indicates the change in the contact region of the humerus with the glenoid cavity rim in the resting position to the abduction and external rotation (ABER) position.

One of the most crucial concepts in HS lesions is the glenoid track (GT), that is, the area of contact between the glenoid cavity and the humeral head during movement from the neutral position to the abduction and external rotation (ABER) position. 20 In this movement, the area of contact moves from inferomedial to superolateral in the humeral head ( Fig. 7 ). Cadaveric studies have shown that the area covered by the glenoid cavity corresponds to 84%, while clinical studies indicate an 83% of coverage. 19

We can use the contralateral side of the glenoid cavity to assess the size of the bone defect; however, this assessment must be careful, as 8% of glenoid cavities present a difference ≥ 3 mm regarding the contralateral side.

The Di Giacomo et al. 9 method for GT measuring includes four steps:

1. Measurement of the diameter of the inferior glenoid cavity using a perfect circle (D);
2. Measurement of the anterior bone loss of the glenoid cavity (d);
3. Calculation of the GT width = (0.83 × D) − d;
4. Measurement of the width of the HS interval (HSI) = width of the HS + width of the bone bridge (BB).

If HSI > GT, the HS lesion is off-track, that is, with a risk of engagement.

**Fig. 8:**
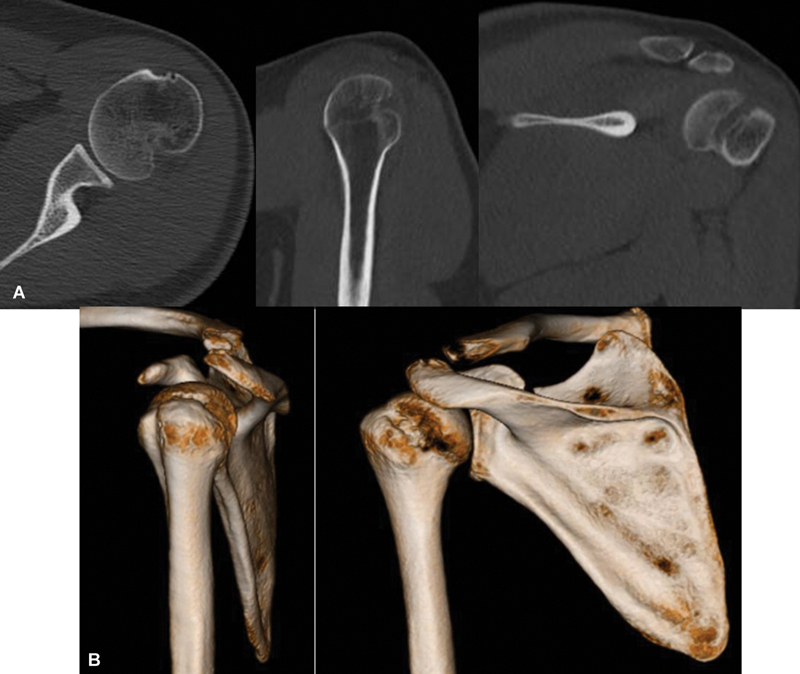
( **A** ) On-track lesion (Hill-Sachs interval [HSI] lesion < glenoid track [GT]); ( **B** ) off-track lesion (HSI > GT).

If GT > HSI, the HS lesion is on track, that is, with no risk of engagement 17 ( Fig. 8 ).

Although the use of this method is frequent in the clinical practice, its intra- and interobserver agreements are low, mainly due to the difficulty in defining the medial margin of the HS lesion, the correct rotator cuff attachment on CT, and the overlap of the lateral edge of the HS lesion on the medial edge of the rotator cuff attachment. 18 19 Another criticism to the GT method is that it does not consider joint mobility, especially in subjects with ligamentous laxity, which may result in higher humeral head excursion. 21

**Fig. 9:**
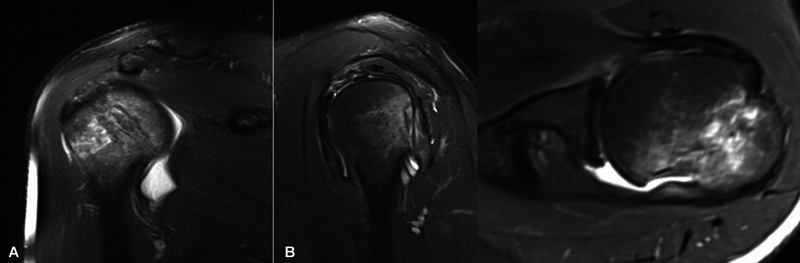
( **A** ) Central track (0 %to 75% of the GT); ( **B** ) Peripheral track (75–100% of GT).

Another imaging classification relies on the location of the HS defect, which was developed from the observation that even some on-track lesions submitted to the Bankart surgery alone evolved with failure. This classification divides the humeral head GT into four zones, and HS lesions reaching the most medial peripheral zone (peripheral track) presented 19 worse results in the Western Ontario Shoulder Instability Index (WOSI) score when compared with other zones (central track) ( Fig. 9 ).

Other measurement methods are under development to reduce failures in on-track lesions treated with labral repair alone. The distance to dislocation (DTD) 22 considers the inferior craniocaudal extension of the HS lesion. 5 Another method is the global track, 21 which uses the dome of the humeral head and its central point as a reference instead of the rotator cuff attachment to measure the HS defect. However, these methods still require validation and testing in the clinical practice.

## Treatment

The treatment of anterior shoulder instability, which is frequently associated with HS lesions, can be conservative in the first dislocation episode in patients with low demand. This treatment strengthens the deltoid, rotator cuff, and scapular stabilizer muscles. It is important to be aware of risk factors for recurrence, such as age (under 20 to 25 years), male sex, epilepsy, fall risk, ligament laxity, and participation in activities requiring ABER or competitive sports. 7 23 24

In cases with surgical indication, HS lesion treatment is not usually performed in isolation, since the labral or ligament complex lesion is virtually always present, and its association with the bone defect of the glenoid cavity (bipolar lesion) occurs in 63% of the cases. 7

The presence of the HS lesion is essential for therapeutic decision-making, as its size, location, and inclination influence the treatment. Some authors 7 25 argue that patients with small bone defects (< 20% of the humeral head) or no engagement can undergo isolated labral repair. Cases of larger defects or engagement may require other procedures. As some cases of failure occur even in the absence of engagement, there is a growing tendency to address the HS lesion in patients with anterior instability, even in situations with borderline bone lesions, especially with the remplissage technique. 21 22 26

## Humeral Head Procedures

The remplissage technique was described in 1972 and comes from the French word for “filling”. Wolf modified it to perform it arthroscopically, “filling” the HS lesion by capsulodesis and infraspinatus tenodesis with labral repair. The prevalence of off-track lesions is of approximately 7%. 27 The ability to convert the HS lesion from intra-articular to extra-articular, reducing the engagement with the inferior edge of the glenoid cavity and the recurrent subluxation rate, determines the success of this technique. Even though the current recurrence rate ranges from 0% to 10%, 28 29 the size of the bone defect in the glenoid cavity influences these values.

The advantage of this procedure is its minimally-invasive approach, avoiding the need for bone block surgeries in the glenoid cavity and its complications. The theoretical disadvantage of this technique is the alteration of the rotator cuff anatomy and the shoulder biomechanics, potentially reducing lateral rotation and causing pain in the posterosuperior region of the shoulder. 30 However, this is a controversial topic, with some studies demonstrating no difference in these outcomes compared with the isolated labral repair technique, especially with the technical development and studies on the ideal location for anchor insertion.

Other options for HS lesion treatment include two procedures: humeroplasty (for acute defects) and osteochondral bone grafting.

Humeroplasty (elevation of the impinged fracture and support with a graft) restores the geometry of the humeral head with no internal fixation. It is indicated for acute injuries of up to 3 weeks presenting less than 40% articular surface involvement. 3

This method consists of elevating the cartilage and filling the defect with calcium phosphate, 31 restoring the local anatomy with a 5° gain in lateral rotation. Another way to elevate the defect uses a percutaneous vertebroplasty balloon with potential videoarthroscopy assistance, 32 33 with report demonstrating a 99.3% reduction in HS lesion bone defect. 7

Partial arthroplasty using allograft fills the defect with an osteochondral graft via an open or arthroscopic approach. Allografts provide better biomechanics restoration, unlike non-anatomical grafts. The potential disadvantages include disease transmission, procedural difficulty, graft reabsorption or failure, subluxation, and cyst formation. 7 In addition, differences in the size and geometry of the defect and the implant may require humeral cartilage milling.

## Glenoid Cavity Procedures

The treatment of glenoid cavity injuries depends on the size of the bone defect. Currently, Bankart repair alone has limited indications. The literature 34 has shown an increasingly guarded prognosis regarding critical bone loss for the performance of the Bankart repair. Adding remplissage to the Bankart repair may reduce the number of surgical treatment failures. 23 35

Bone procedures increase the surface area of the glenoid cavity and indirectly treat potential failures resulting from the HS lesion. The most widespread techniques are the Latarjet procedure, the Eden-Hybinette technique, and the tibial allograft.

The potential disadvantages of the bone graft techniques in the glenoid cavity are loss of lateral rotation (on average, 11°), infection, hematoma, graft resorption, pseudoarthrosis or fibrous union, and subscapularis injury. 23 30

## Arthroplasties

Partial resurfacing prostheses present the advantages of no donor site morbidity compared with autografts and shorter surgical time. The disadvantages are the difficulty in obtaining proper fixation and the inability to align the prosthesis surface with the humeral articular surface. 36

Another option is hemiarthroplasty, a procedure indicated for elderly patients with an HS defect > 40% of the articular surface. An increase of 10° to 15° in retroversion may improve stability. 7 25

Other arthroplasty types are uncommon and intended for more severe instability or complications from previous procedures.

## Options with a Historical Interest

Weber's derotation osteotomy is a treatment option still in use as a salvage procedure in young patients. This technique consists of an osteotomy on the surgical neck of the humerus and a retroversion of the humeral head in relation to the diaphysis. This procedure presents variable outcomes and relatively high complication rates, such as pseudarthrosis, iatrogenic fracture, and osteoarthritis. 7

## Final Considerations

The HS lesion plays a fundamental role in shoulder instability, and research on this topic has exponentially increased in recent times.

The search for an algorithm for decision-making on the best treatment for patients with anterior shoulder instability continues. However, despite all attempts and concepts developed to date, there is no ideal option.

Therefore, it is essential to consider HS lesions and their correct treatment to improve outcomes and achieve high success rates, with no dislocation recurrence and excellent function according to patients' expectations. We expect that the evolution we are witnessing will bring long-term better functional outcomes and fewer complications to these patients who, as already discussed, are mostly young, active people, with high expectations.
